# Mirabegron improves the irritative symptoms caused by BCG immunotherapy after transurethral resection of bladder tumors

**DOI:** 10.1002/cam4.4278

**Published:** 2021-09-21

**Authors:** Kai Sun, Di Wang, Gang Wu, Jian Ma, Tianqi Wang, Jitao Wu, Jipeng Wang

**Affiliations:** ^1^ Department of Urology The Affiliated Yantai Yuhuangding Hospital of Qingdao University Yantai Shandong China

**Keywords:** BCG immunotherapy, irritative symptoms, mirabegron

## Abstract

**Background:**

This study aims to explore the efficacy and safety of mirabegron in treating irritative symptoms induced by intravesical immunotherapy with Bacillus Calmette–Guerin (BCG) after transurethral resection of bladder tumors (TURBT).

**Methods:**

A total of 160 patients subjected to TURBT was randomly divided into the mirabegron group and placebo group with 80 patients in each group. Then, the patients were administered 25 mg mirabegron or placebo daily, starting the first day after BCG infusion. The first BCG perfusion was conducted at least 2 weeks after TURBT. The 3‐day bladder diaries were completed in all patients, 1 day before BCG perfusion, and on the 1st, 6th, and 13th days after the first BCG perfusion. Overactive bladder symptom scores were completed 1 day before BCG perfusion, and on the 6th and 13th days after the first BCG perfusion.

**Results:**

Symptom scores of bladder hyperactivity were significantly different between the two groups (*p* < 0.001). Also, the frequency of nocturia, pollakiuria, micturition urgency, urinary incontinence and was significantly lower in group 1 than that in group two (*p* < 0.05).

**Conclusion:**

Our findings demonstrate that mirabegron is a valuable clinical drug for the management of irritative symptoms after TURBT with subsequent intravesical BCG perfusion.

## Graphical abstract

BCG infusion after TURBT may cause urinary tract irritation. Mirabegron showed significant relief of symptoms compared to placebo.

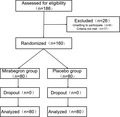

## 1 INTRODUCTION

Bladder cancer (BC) is the ninth most commonly diagnosed cancer across the globe.^1^ The current diagnostic criteria of BC rely on a combination of cystoscopy, voiding cytology, and imaging.^2^ Research approximates that 70%–80% of patients are categorized as non‐muscle invasive bladder cancer (NMIBC) during the initial stages of diagnosis^3^; where, the transurethral resection of bladder tumor (TURBT) is used as the first‐line therapy.^4^ Patients with NMIBC have an extremely high recurrence rates of 65%, 81%, and 88% for bladder cancer at 5‐, 10‐, and 15‐years follow‐up after TURBT, respectively. Some patients may progress to muscle‐invasive bladder cancer (MIBC).^1^ Therefore, some intravesical instillation agents have been used as complementary therapy after TURBT. Perfusion chemotherapy represented by adriamycin (ADM), mitomycin (MMC) and epirubicin (EPI), and bladder perfusion immunotherapy represented by Bacillus Calmette–Guerin (BCG) have shown good efficacy in preventing recurrence. Compared with other intravesical agents, intravesical BCG has been reported to significantly prolong the time to first recurrence and also reduce the risk of progression to MIBC.^5^ Yuk et al. demonstrated that patients who receives BCG maintenance therapy reaches T0 after multiple TURBT and have significantly fewer tumor recurrences compared with the patients who received no BCG treatment or only BCG induction therapy.^6^ Therefore, intravesical BCG has been considered to be the standard treatment for patients with intermediate or high risk of NMIBC after TURBT and has been widely used in clinical practice.^7^

While acknowledging the significant contributions of BCG to the reduction of recurrence and progression, severe side effects have been reported causing pain to the patients.^8^, ^9^ This leads to poor compliance with BCG treatment. Complications caused by BCG include local and systemic side effects and symptoms might range from self‐limiting irritant urination to severe systemic sepsis.^10^, ^11^ Frequency, urgency, painful urination, hematuria are the most prevalent symptoms of local side effects, occasionally accompanied by acute urinary incontinence, somewhat analogous to overactive bladder (OAB).^12^ It is primarily manifested as catheter‐related bladder discomfort (CRBD) with the incidence approaching 90% after TURBT.^13^ Additionally, with the extension of BCG treatment, the incidence and severity of the condition with adverse reactions (AE) also increase. A study that included 1316 patients treated with BCG reported that chemical cystitis occurred in 35% of the patients whereas 103 patients discontinued the treatment due to complications.^14^ Elsewhere, 19% of patients receiving intravesical BCG have been reported to discontinue treatment due to complications whereas only 29% completed the immunotherapy for 3 years.^8^ Therefore, immediately and effectively controlling the symptoms improve compliance by the patients, and ameliorates quality of life.

The detrusor muscle in humans is relaxed by β3‐adrenoreceptor‐mediated sympathetic activation, which can flatten and relax the bladder hence promote urine storage.^15^ Mirabegron is a newly developed β3‐adrenoreceptor agonist, constituting the emerging pharmacotherapy strategies of managing OAB symptoms.^15^, ^16^ It has gradually become a more popular clinical drug for the treatment of OAB because of its similar efficacy but fewer side effects compared with the most commonly used muscarinic receptor antagonists.^17^ Mirabegron has demonstrated significant efficacy and safety in the treatment of lower urinary tract symptoms (LUTs) following stress urinary incontinence surgery in women and OAB due to benign prostatic hyperplasia with bladder outlet obstruction in men.^18^, ^19^ However, its efficacy against symptoms of local side effects after TURBT with subsequent BCG immunotherapy remains unexplored.

Therefore, this work compares mirabegron‐treated with placebo‐treated counterparts to ascertain the efficacy and safety of mirabegron against the local side effects of BCG therapy after TURBT.

## 2 MATERIALS AND METHODS

This is a randomized, double‐blind, placebo‐controlled, prospective clinical trial research of mirabegron conducted by the Department of Urology of Yuhuangding Hospital over 8 months. The crossover design was not applied to this study. Based on the principles of the Helsinki Declaration, the study was approved by the institutional ethics committee (approval number is 2017–208). The clinical trial registration number was ChiCTR2000040823, which was approved by the Chinese Clinical Trial Registry. All the participants were comprehensively informed of the risks associated with the research and they provided their informed consent for participation.

Patients aged 18–80 years with pathological diagnoses of intermediate‐risk and high‐risk NMIBC were included. All patients with bladder stones, glaucoma, obstruction of the bladder outlet, previous pelvic irradiation treatment, urinary tract infections, morbid obesity, chronic pain, chemical substance abuse, severe renal, or heart diseases, those who had taken or under drugs beneficial to OAB were excluded. Moreover, considering the high likelihood of patients developing OAB after TURBT due to indwelling catheters, patients with a previous diagnosis of OAB before BCG perfusion were excluded.

The randomization method is indicated below: The participants were arranged in numbers from 1 to 160 based on the chronological order in which they engaged in the trial from August 2017 to August 2019. SPSS version 26 was used to generate 160 random numbers, which were rearranged into group 1 and group 2, with 80 patients in each group. Based on European Association of Urology (EAU) guidelines, BCG therapy was administered once a week for 6 weeks to trigger an immune response, plus increased three doses at a weekly interval in the 3rd, 6th, and 12th months to maintain a good immune response. The first BCG perfusion was conducted at least 2 weeks after TURBT. The ImmuCyst^®^ (1.8 to 19.2 *10^8^ cfu or 81 mg Connaught strain BCG) was selected for perfusion therapy. After receiving a similar dose of treatment with BCG perfusion, group 1 patients were administered with 25 mg/d mirabegron for 2 weeks. Considering the more severe symptoms of irritation caused by BCG, mirabegron 50 mg/d was administered from the 3rd week until the end of the induction period. In group 2, patients obtained a placebo instead of mirabegron. By sight, smell, and taste, it was difficult to spot the differences between drugs and placebo.

All patients received antibiotics from postoperative to 3 days after the removal of the catheter. Overactive bladder (OAB) symptom score (OABSS)^20^ and 3‐day bladder diaries were completed before bladder perfusion to obtain baseline values. Patients self‐recorded the side effects of BCG perfusion after every infusion. AE to mirabegron have also been documented. The patients completed OABSS on the 6th and 13th days after the first BCG perfusion and recorded the frequency of nocturia, pollakiuria, micturition urgency, hematuria, urinary incontinence, and odynuria on the 1st, 6th, 13th days after the first BCG perfusion. Urine analysis and culture were performed 3 days before and 3 days after each bladder perfusion to prevent the effect of bladder irritation caused by bacterial urinary tract infection. Patients diagnosed with urinary tract infection were withdrawn from the study and received symptomatic treatment. Based on severity of the AE suffered by the patients, the physicians decided whether to proceed, postpone, explicitly delete, or delete the next perfusion, or whether the patient should be treated with antituberculosis therapy. Cystoscopy, biopsy, and urinary cytology were performed to identify the efficacy of BCG therapy every 6 months during the first year.

Based on the OABSS published standard deviation of 3.0^21^ and a bilateral alpha value of 0.05, 127 participants needed 90% statistical power to detect OABSS differences between the two groups; the number increased to 160 to account for potential dropouts.

Statistical analyses were conducted using SPSS26.0 (IBM). Two‐way repeated‐measures ANOVA was used to analyze the changes in the efficacy parameters of patients in the two groups from the initial diagnosis to the final diagnosis, then the adjusted mean value and standard deviations (SD) were obtained. A *p* value of <0.05 (*p* < 0.05) was considered significant.

## 3 RESULTS

**FIGURE 1:**
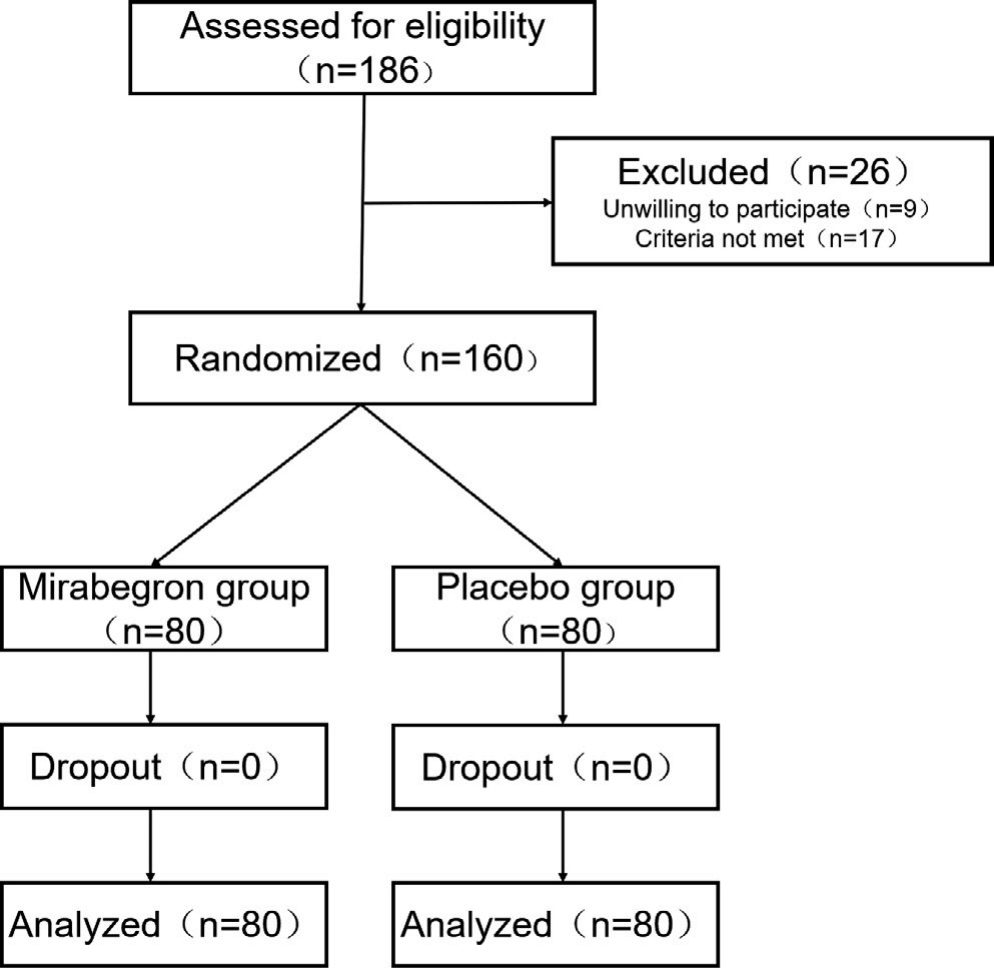
Flow chart of patient enrolment and randomization

In total, 186 patients were evaluated, among them, 26 were excluded for not meeting inclusion criteria or unwilling to participate. Thus, only 160 patients were enrolled. They were randomly divided into two groups (mirabegron and placebo groups) and administered with study medication. Although mild to moderate AE occurred during treatment, no participants dropped out or lost to follow‐up; all of them completed the study (80 in each group; Figure 1). Regarding tumor aggressiveness, there was no significant difference between the two groups. Intermediate to high risk and superficial bladder cancer was noted in the majority of included samples. The tumor‐related and patient‐related characteristics are illustrated in Table 1. In general, both the two groups had similar patient demographics and baseline characteristics.

**TABLE 1:** Baseline characteristics in mirabegron group and placebo group

	Mirabegron group (*n* = 80)	Placebo group (*n* = 80)
Age (y)	60.91 ± 5.06	61.98 ± 5.02
Sex radio (M:F)	43:37	41:39
Stage of bladder tumor: Tis:Ta:T1	8:25:47	7:21:52
Histologic grade of bladder tumor:	19:37:24	20:35:25
G1:G2:G3	18:35:27	19:33:28

In terms of efficacy parameters, the OABSS and the frequency of nocturia, pollakiuria, micturition urgency, hematuria, urinary incontinence, and odynuria before operation and BCG perfusion showed no significant difference between the two groups. However, it was observed that the OABSS significantly decreased in the experimental group on the 6th (*p* < 0.001) and 13th day (*p* < 0.001). As for the frequency of nocturia, no observable difference was noted between the two groups on the 1st day (0.63), whereas, there was a significant difference between the 6th (*p* < 0.001) and the 13th day (*p* = 0.03). Regarding the frequency of pollakiuria and micturition urgency, a notable difference emerged between the two groups in the 1st (*p* < 0.001), 6th (*p* < 0.001), and 13th (*p* < 0.001) day, respectively. However, in terms of the frequency of hematuria, the experimental group did not enjoy superiority to the control group in the 1st (*p* = 0.75), 6th (*p* = 0.70), and 13th (*p* = 0.65) day. Besides, the mirabegron group was dominant in the frequency of urinary incontinence compared to the placebo group in the 1st (*p* < 0.001), 6th (*p* = 0.01), and 13th (*p* = 0.01) day. The results are comprehensively shown in Table 2.

**TABLE 2:** Results of OABSS and bladder diary

	Mirabegron group (mean ± SD)	Placebo group (mean ± SD)	*p* value
OABSS			
Pre‐prefusion	2.40 ± 0.49	2.45 ± 0.50	0.53
6th day	5.48 ± 0.59	6.74 ± 0.67	<0.001
13th day	3.98 ± 0.62	5.24 ± 0.64	<0.001
Nocturia			
Pre‐perfusion	0.39 ± 0.49	0.43 ± 0.50	0.63
1st day	1.90 ± 0.54	1.86 ± 0.55	0.66
6th day	0.75 ± 0.49	1.90 ± 0.52	<0.001
13th day	0.53 ± 0.50	0.75 ± 0.51	0.03
Pollakiuria			
Pre‐perfusion	6.58 ± 0.69	6.68 ± 0.71	0.367
1st day	9.01 ± 0.75	11.81 ± 0.89	<0.001
6th day	8.34 ± 0.53	10.68 ± 0.92	<0.001
13th day	7.53 ± 0.50	9.04 ± 0.82	<0.001
Micturition urgency			
Pre‐perfusion	0.41 ± 0.50	0.49 ± 0.50	0.34
1st day	2.10 ± 0.63	3.34 ± 0.62	<0.001
6th day	1.66 ± 0.50	2.48 ± 0.50	<0.001
13th day	0.45 ± 0.50	0.73 ± 0.57	<0.001
Hematuria			
Pre‐perfusion	0.63 ± 0.24	0.88 ± 0.28	0.55
1st day	0.40 ± 0.49	0.43 ± 0.50	0.75
6th day	0.19 ± 0.39	0.21 ± 0.41	0.7
13th day	0.13 ± 0.33	0.15 ± 0.36	0.65
Urinary incontinence			
Pre‐perfusion	0.35 ± 0.48	0.33 ± 0.47	0.74
1st day	0.94 ± 0.62	1.35 ± 0.62	<0.001
6th day	0.49 ± 0.50	0.75 ± 0.44	0.01
13th day	0.19 ± 0.39	0.36 ± 0.48	0.01
Dysuria			
Pre‐perfusion	0.11 ± 0.32	0.14 ± 0.35	0.64
1st day	4.75 ± 0.57	4.88 ± 0.68	0.21
6th day	3.85 ± 0.51	3.88 ± 0.68	0.79
13th day	2.18 ± 0.59	2.30 ± 0.49	0.15

Additionally, the adverse events were recorded to assess the safety and toleration of mirabegron. The number of AE was carefully counted in the mirabegron group, including two cases (2.5%) of gastrointestinal symptoms, two cases (2.5%) of rash, one case (1.25%) of hypertension, one case (1.25%) of elevated PVR (more than 200 ml), one case (1.25%) of palpitation, and one case (1.25%) of constipation. Fortunately, these symptoms were seemingly not severe and the side effects were tolerable. In the placebo group, we found six patients with eight adverse events; after deliberations by two professional doctors, it was concluded that these adverse events were not related to the study. Table 3 illustrates the specific results of the AE.

**TABLE 3:** Occurrence of adverse reactions in the mirabegron group

Adverse reaction	Mirabegron group no. (%)
Gastrointestinal symptom	2 (2.5)
Rash	2 (2.5)
Hypertension	1 (1.25)
Elevate PVR (more than 200 ml)	1 (1.25)
Palpitation	1 (1.25)
Constipation	1 (1.25)

## 4 DISCUSSION

Intermediate and high‐risk NMIBCs are commonly performed by TURBT, followed by BCG immunotherapy to prevent recurrence and progression of tumors. Five meta‐analyses unanimously reported that BCG is more effective in preventing recurrence after TURBT surgery than TURBT alone or TURBT plus chemotherapy.^22^, ^23^, ^24^, ^25^, ^26^ BCG does not directly poison tumor cells, instead, it generates a series of immune responses that trigger the destruction of the tumor. Nonetheless, since the use of BCG causes local and distant inflammatory responses, the local and systemic side effects have significantly increased.^27^, ^28^

This study found that 25 mg mirabegron is markedly beneficial to patients with irritation who have experienced TURBT with subsequent BCG perfusion. Specifically, it improves OABSS and the frequency of nocturia, pollakiuria, micturition urgency, and urinary incontinence. Besides, the chemical cystitis symptoms caused by BCG correlated to those of OAB. Notably, the symptoms of urination have been the most direct and painful complication of BCG perfusion. Therefore, the ability to manage these symptoms is fundamental to efficacy assessment. OABSS is an important tool for the diagnosis of OAB and the assessment of OAB symptoms. The effect of mirabegron which is a novel drug for the treatment of OAB, in reducing OABSS in this study was in line with study expectations. Nocturia, pollakiuria, micturition urgency, and urinary incontinence are the symptoms caused by BCG perfusion, which are the most direct reasons for reducing patient compliance. By improving the above symptoms, mirabegron can relieve pain and improve the compliance of patients as well as greatly improves the prognosis of patients.

Various measures have been taken in response to the intense inflammatory reactions caused by BCG perfusion. Reducing the perfusion dose and increasing the perfusion interval are considered effective measures. However, these may increase the risk of incomplete treatment due to discontinuation of the drug.^29^ Isoniazid, a tuberculostatic agent, have also been investigated for prophylactic use with each BCG perfusion to reduce the incidence of AE.^30^ It was found that isoniazid with BCG had no difference in AE compared with BCG alone and was associated with more liver toxicity. Ofloxacin, a fluoroquinolone with antituberculosis capacity, was another approach for decreasing AEs. Colombel et al. performed a randomized trial and found that ofloxacin significantly reduces the incidence of moderate and severe AEs to BCG perfusion.^31^ However, further studies are needed to identify that ofloxacin does not affect the antitumor effect of BCG. Although the above methods partially alleviated AEs and improved patient compliance, the antitumor effect of BCG was also impaired. Mirabegron significantly alleviates the irritative symptoms caused by BCG perfusion without affecting the dose and drug properties of BCG and can greatly preserve the antitumor capacity of BCG. These are the prominent contributions of mirabegron compared with the above regimen.

Solifenacin, a muscarinic antagonist used to treat OAB was used to treat irritative symptoms in patients undergoing TURBT with succeeding intravesical chemotherapy.^32^ Consequently, solifenacin significantly improved urinary frequency and incontinence, as well as bladder irritation. Based on recent studies, mirabegron appeared to have more advantages than solifenacin in OAB treatment. Random Controlled Trials (RCTs) in Europe, Australia, Canada, and the United States have demonstrated the efficacy and tolerability of mirabegron.^16^, ^33^ In this study, the average number of incontinence and frequency of urination from baseline to final visit was significantly improved in mirabegron patients compared to placebo. The treatment effect between mirabegron and solifenacin showed no significant difference, however, AE, including dry mouth and constipation of mirabegron was significantly higher than that of solifenacin.

Mirabegron, the first β3‐adrenoreceptor agonist in clinical use, effectively resolves the shortcoming of anti‐muscarinic drugs. Adrenoreceptor in bladder detrusor cells and urinary epithelial cells has three subtypes, including 1, 2, and 3, where 3 receptor mRNA is primarily expressed in detrusor cells, accounting for 97% of the total receptor mRNA.^34^ Thus, mirabegron relaxes the detrusor by activating the three adrenoreceptor, increasing bladder volume, and prolonging the interval between two urination sessions without affecting urination activity during the urination phase.^35^ Conventionally, the molecular mechanism by which β3‐adrenoreceptor agonist inhibits spontaneous contraction is thought to be via activation of second messenger cAMP which reactivates PKA. Moreover, PKA phosphorylates vital intracellular target proteins and ultimately causes smooth muscle relaxation. Nonetheless, Petkov discovered that the role of cAMP in mediating bladder relaxation is remarkably less than that of the K^+^ channel.^36^ Elsewhere, Michel et al. found that, unlike anti‐muscarinic drugs, β3‐adrenoreceptor agonist did not influence the pressure and residual urine volume during the urination period, while it increased the bladder volume and reduced the number of urinations.

Herein, mirabegron revealed an insignificant advantage in improving hematuria and odynuria symptoms. However, this did not affect the compliance with bladder perfusion; since, unlike hematuria caused by bladder tumors, hematuria induced by immunotherapy is non‐persistent and does not cause moderate or higher levels of anemia.^37^

Mirabegron was effectively tolerated and gastrointestinal symptoms and rash, which in most cases was mild were the most prevalent AE. Notably, one patient developed hypertension and palpitations after using mirabegron, and researchers attributed it to the treatment because the patient had no history of the disease. Mete et al. reported that symptoms including hypertension and palpitations can be relieved after withdrawal of the drug.^18^ The quality of life among patients with other AE remained unaffected.

This study was the first to apply mirabegron to the treatment of urinary tract irritative symptoms caused by bladder perfusion with BCG after TURBT. It was shown that, although there was no favorable benefit of mirabegron in improving hematuria and odynuria, mirabegron significantly relieved nocturia, pollakiuria, micturition urgency, and urinary incontinence. Further, mirabegron improved the symptoms of urinary irritation without compromising the antitumor effects of BCG. This significantly relieved patient from suffering, increased patient compliance to treatment and contributed to patient completion of the entire BCG perfusion course. This study provides a good option for the treatment of urinary tract irritation in patients who are about to apply or are applying BCG perfusion for NMIBC after TURBT. It also enlightened us that mirabegron may also have a better role in urinary tract irritation symptoms that occur during bladder perfusion chemotherapy. This study has worth‐mentioning limitations. First, consistent with most BCG studies, the interval between tumor resection and the first perfusion was limited to not less than 2 weeks. Thus, it was difficult to solely attribute local symptoms to BCG perfusion. Second, all the participants were of Asian and Chinese origin; outcomes in other races and countries were not assessed. Third, this trial used a small sample size, therefore, a larger study is warranted. Fourth, we measured the frequency rather than the degree to evaluate odynuria, consequently, this hampered the observation of a more intuitive improvement. Fifth, the detrusor activity index, a suitable diagnostic model for OAB evaluation with high accuracy was not used.

## 5 CONCLUSION

In conclusion, BCG perfusion therapy remains the gold standard treatment option for NMIBC after TURBT; the resulting bladder irritation symptom is presently the primary factor influencing the compliance of patients and therapeutic effect. In this regard, our findings reveal that mirabegron is a valuable and safe drug for the management of these irritant symptoms.

## CONFLICTS OF INTEREST

The authors have no conflict of interest relevant to this article.

## CONSENT FOR PUBLICATION

All authors agree to publish this article.

## ETHICAL APPROVAL STATEMENT

The crossover design was not applied to this study. Based on the principles of the Helsinki Declaration, the study was approved by the institutional ethics committee (approval number is 2017–208).

## CLINICAL TRIAL REGISTRATION NUMBER

The clinical trial registration number was ChiCTR2000040823, which was approved by the Chinese Clinical Trial Registry.

## Data Availability

The data that support the findings of this study are available from the corresponding author upon reasonable request.
